# Power of narrative: a case study about documenting private insightful experiences while dealing with pain and associated disability

**DOI:** 10.3389/fdgth.2023.1289373

**Published:** 2023-12-22

**Authors:** S. F. Lakha, S. F. Sohail, C. B. Holtzman, Z. A. Akkok, A. Khandwala, W. Suhanic, P. Pennefather, D. I. Fels

**Affiliations:** ^1^ Inclusive Media and Design Centre, Ted Rogers School of information Technology Management, Toronto Metropolitan University, Toronto, ON, Canada; ^2^ Institute of Medical Sciences, Temerty Faculty of Medicine, University of Toronto, Toronto, ON, Canada; ^3^ School of Administrative Studies, York University, Toronto, ON, Canada; ^4^ gDial Inc., Toronto, ON, Canada

**Keywords:** chronic pain, audio-video recordings, digital data, personal health record, qualitative evidence

## Abstract

**Objective:**

People adjusting to living with a chronic disability, such as chronic pain, seek support and resources from societal systems, including health systems, to help them cope with this reality. This case study describes the use of a digital health platform designed to help in that quest.

**Method:**

MyHealthMyRecord (MHMR), is being developed to record, register and curate personal private experiences of a chronic condition. MHMR allows users to record and log short (30–90s) personal and private audio-videos of their accommodation-seeking journey in a way that can be encrypted, registered, curated and shared privately. This case study describes the use of a prototype version of the platform by a participant co-designer who experienced a sudden onset of a chronic pain condition, of undetermined origin. System use began three months after the onset of the condition and just after being discharged from several months of hospitalization without any definitive diagnosis.

**Result:**

During a three-month period, 65 short unstructured contributions were authored and logged. This paper presents a qualitative analysis of that content. The clips used various communication styles that documented experiences, concerns, issues, positive and negative interactions and pain episodes. Using thematic analysis with open coding, three domains (person-facing, accessibility and system-facing) and eight themes (pain, joy, therapy, environmental, recommendations, technical, culture and communication) were identified. Comments about pain, stress, etc., were the most common and occurred in 75% of all videos while technical and therapy/physio related comments were the fewest and occurred in 3 and 9% of the videos, respectively.

**Conclusion:**

We conclude that it is possible to create recordings of events, thoughts, reflections and issues on different aspects affecting an individual's health and well-being impact, including effects of the chronic condition as well as tangential outcomes such as accessibility (or lack of it), using MHMR over a longer period of time. The next steps will be to develop functionality to annotate the recordings, automatically analyze and summarize collections of recordings to make them consumable, useful and understandable to the individual and others, and then to share those analyses and summaries with others. In addition, evaluate this functionality longitudinally with more users.

## Introduction

People who suffer the sudden onset of a disabling chronic condition experience a shock to their routine and a disruption of their life-course, as well as to their medical and social situations. They need support in learning to cope and engage with making sense of their new reality (1). That support can take the form of therapeutic/clinical medications and casual/formal social practices involving both professional support from counsellors and support workers and social support from a broader circle of care. Living with chronic conditions typically involves active negotiation of episodically disabling situations (2) in a manner that requires active engagement by the affected person rather than just their passive acceptance of recommended coping strategies and simple compliance with imposed therapeutic/rehabilitation interventions (3). That engagement requires communication of evidence of private, personal needs and wants to require accommodations in order to advocate for accommodations that often are mandated by law or institutional policies (3, 4).

Increasingly, that communication involves digital technology. However, human factors considerations such as diverse orientations and false expectations have been shown to limit positive effects from implementing digital health technology for supporting person-centred approaches and self-advocacy (4). Person-centred approaches to healthcare are intended to promote agency, engagement, empowerment and self-efficacy in the person receiving the care (1, 5). We propose that having a patient register their experiences with care, technologies, and interventions can lead to the development of important person-centred healthcare aims of autonomy and independence as discussed by (6).

We are developing a system for documenting, registering, and sharing human factors such as agency, engagement, empowerment and self-efficacy by users of healthcare, rehabilitation and accessibility programs. This is carried out through enabling the generation of a personally owned and curated digital repository (“scrapbook”) of short video recordings of personal observations concerning an individual's care/recovery/accommodation journey following the sudden onset of a chronic condition. We believe that the video nature of this record will make it more accessible and meaningful to the author and those with whom they decide to share their record(s). It will also set the stage for creating a corpus of voice/video evidence for directing care that are generated and owned by patients and are ready for regulated precision reporting regimes.

The MyHealthMyRecord (MHMR) platform is designed to document, register and share a person's private experiences with a chronic condition for which they seek care, support, and accommodations (7, 8). The idea is to allow them to assert ownership over person-centered evidence used to guide interventions designed to assist them in coping with their condition.

The aim of this case study was to explore how digital scrapbooking of short audio-video (AV) commentaries may offer an accessible and inclusive method for recording and anchoring person-centred documentation of their personal healthcare journeys. The MHMR platform described here is based on video guestbook technology developed earlier to help people living with disabilities to comment on hospitality services they experienced (9). MHMR is being designed around the concept of curating a digital scrapbook made up of an unstructured collection of short-duration, first-person AV's that register commentary on concerns, issues, successes, and failures of care and support the user has experienced. Although designed first to be a tool for self-reflection, we are confident that with time, user-curated repositories of such AVs can be used to create a registered representation of the patient's voice useful more generally in advocating for their needs and wants as people with lived/living experiences, and those of others like them. MHMR technology is designed to highlight the user's experience, perspective, and identity as these relate to the care support and accommodations that they access for their chronic condition. The short format makes them amenable to quick review and analysis by both human and machine entities trained to recognize patterns pointing to available solutions. MHMR provides a counter-narrative to the official, system-serving record of care. In this paper we illustrate how the well-known method of thematic analysis of the qualitative MHMR content can be used to structure the unstructured person-reported observations (PROs).

This paper illustrates how the MHMR technology can allow the person receiving support and care for a chronic health condition to express his or her needs, ideas, experiences, concerns, and coping strategies in an easily generated and accessible manner. Such evidence is now recognized as vital to patient self-management necessary for effective community-based care (10). In addition, this paper comments on how such data can fill a need to accommodate human factors such as diverse orientations and false expectations that have been shown to limit positive effects from implementing approaches that seek to register patient experiences (4).

The research questions addressed in this paper are thus: (1) what is the feasibility of recording short-duration first-person perspective narrative reflections on condition-associated challenges? and, (2) what are the experiences, expressions, and attitudes expressed over time using the platform by a participant co-designer whose life was disrupted after the sudden onset chronic pain of undefined causes? In order to preserve the privacy and anonymity of the participant, gender pronouns are alternated in this paper, and no institutional names are provided.

## Background

This literature review provides a brief overview of the need for people-centred applied science and design, highlighting the mostly unexplored potential of qualitative information to enhance communication between individuals with illness and health professionals. It examines individual engagement and empowerment through curated health data to facilitate sharing with health professionals and care circles.

Individuals with chronic illnesses must manage their conditions while also engaging with the healthcare system. While programs exist to assist in medical management and holistic care, there is an apparent paucity of resources and research addressing the thorough tracking, integration, and communication of activities individuals perform to cope with their illnesses between healthcare specialist appointments (11). For example, someone may use naturopathic and medical treatments, but this is not necessarily conveyed during appointments due to reasons such as memory, significance, priorities, embarrassment, and readiness to discuss at the time of their visit.

Despite extensive advocacy for person/patient-centred care by governments, healthcare organisations, and individuals, its implementation has often proven limited, unsustainable, or unsuccessful (12). Additionally, the absence of a reliable and trusted method for registering, recording and tracking patient perspective within the medical record so as to demonstrate benefits of patient involvement in setting care priorities persists. While considerable efforts have been made to measure quantitative patient data, these processes are primarily driven by clinicians' needs (13), but there is a high chance of data abandonment rates (14). While clinicians acknowledge the usefulness of person-centred care, there is no standardized methodology for its implementation or integration. For instance, patients may gain access to their institutional health record items and lab results (15), but this access does not extend to documenting a patient's complete care journey (16).

Ongoing progress and likelihood of future innovations enabled by existing natural language processing system and, data science, promise to enhance communication between patients and healthcare providers in ways that promote voice based sensemaking (8). However, effective communication also requires an understanding of cognitive factors, such as the social embodiment on how information is perceived and communicated (17).

Research and design methodologies derived from human factors, co-design (18), and human-computer communication/interaction paradigms provide a pathway to mobilize diverse perspectives and ways of knowing in the co-creation of actionable meaning, aligning with the WHO Framework on integrated people-centered health services (19). The distinction between person-centered and patient-centered healthcare lies in the contrast between building meaning and building function (20). The evaluation of our tool's impact on increasing acceptability, reducing communication barriers, and enhancing trust between healthcare providers and chronic pain patients is central to our research, contributing to the construction of meaning-in-care. However, it is important to consider the willingness and feasibility of successfully producing and capturing qualitative information on a long-term basis. In addition, what topics and ideas constitute these data must also be assessed to determine whether they can be used in a meaningful way to support documentation, self-assessment, reflection and communication.

The Structure-Process-Output framework is derived from the Donnebedian model of clinical activity (reference) that has been used for more than 50 years. It has been shown to help physicians make sense of and construct a self-narrative about how the clinical care structures they interact with to deliver therapeutic processes can lead to desired clinical outcomes (21). Recently, Lakha et al. (22, 23) have proposed a hybridization of the Donnebedian model, with a more generalized and evaluative “theory-of-change” represented as a logic model (24). The idea of the proposed Donnebedian-Logic Hybrid (DLH) evaluation model is to focus on more proximal consequences of structure and process interactions that can be directly appreciated by the reflective evaluator with respect to expected outputs. Accordingly, a “theory-of-change” or reflections on why the change is, or is not, occurring as expected can be documented and reported as evidence within a cause-and-effect framework. This allows all individuals involved in the care process, including patients and their circle of care, to augment the medical record in a manner that is still meaningful for both patients and doctors while still useful for efficient translation of processes into desired outcomes. This will be a necessary improvement in health system record keeping as it transitions from a clinician-centred approach to a more patient-centred one.

The DLH evaluation model is adapted in this paper to design a way for patients/system-users to gather evidence, in the form of short video recordings of opinions and reflections, for making sense of appreciated or disappointing outputs associated with their ongoing interaction with system structures and processes associated with health system services.

The goal of MHMR is to create a patient-owned repository of experiential evidence organized within a Structure-Process-Output framework (SPO) that can guide the interpretation of this patient generated evidence. The platform is being designed to help the user to document observable model elements of patient-centered Structure/Inputs and Process/Activities, and then process them into observable/actionable Outputs.

### MHMR system design

The MHMR platform is being developed to support collecting and assessing qualitative data generated by patients. It may also be possible in future development to integrate more quantitative data from the health system that is increasingly accessible to patients through existing patient portals (10) and emerging health data-sharing platforms (25).

**Figure 1:**
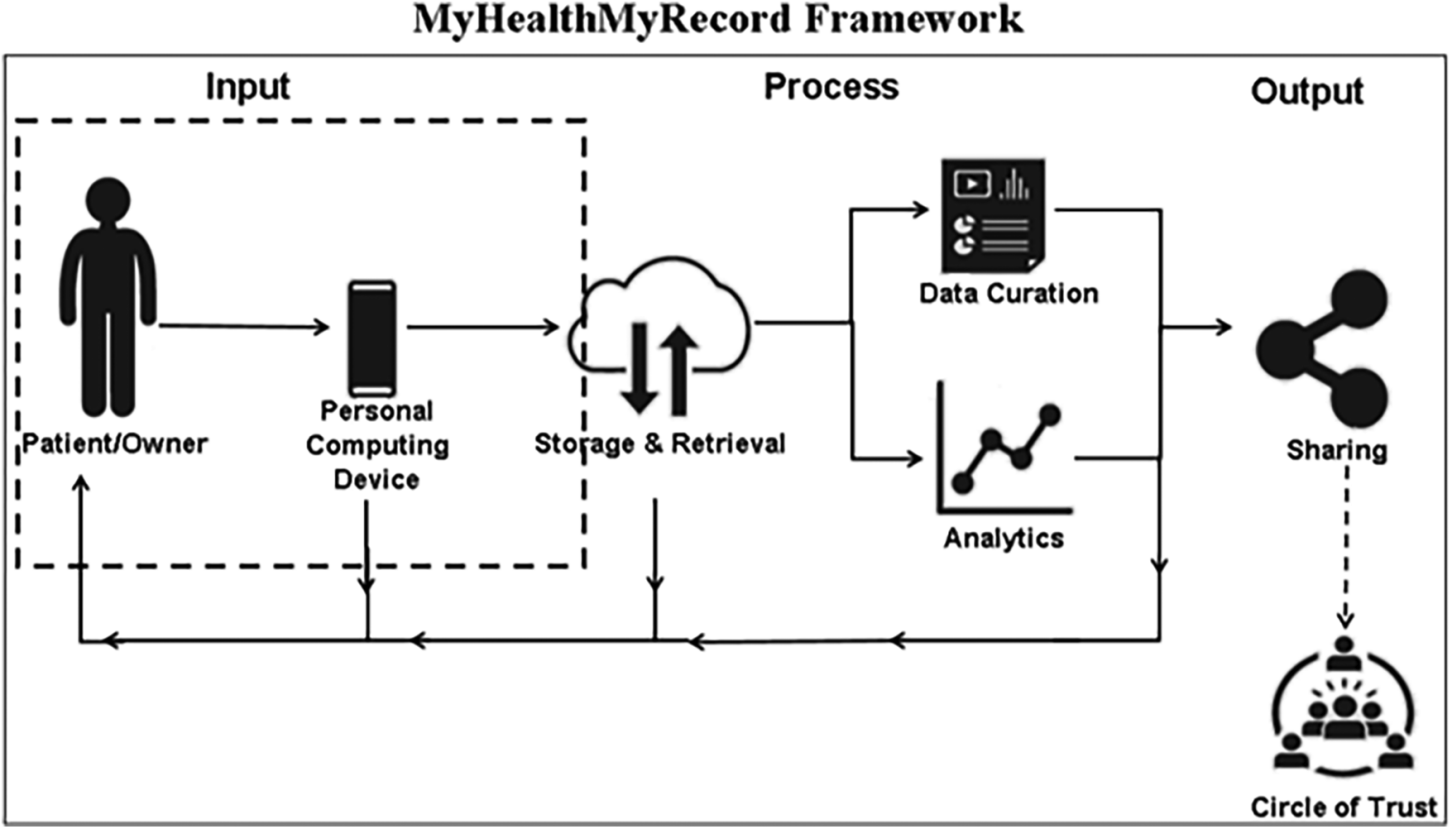
Myhealthmyrecord framework.

The current embodiment of the MHMR platform consists of a tablet-based video recording application that accepts short-duration audio/video user input (Figure 1). The videos are then stored on a server for secure transfer, processing, and storage of video records. In the embodiment reported here, the primary functionality is to serve as a digital video scrapbook which documents and bundles a person's experiences with their disabling condition.

As with the video-guestbook technology from which it was derived (9), the MHMR platform was designed to be inclusive of users with different needs (e.g., sign language users) by allowing them to record short duration audio and/or videos on the topic of their choice at their leisure. The aim was to provide users with a safe way to record, not only their physical state but also their emotional state, without sacrificing their independence or privacy. Privacy was protected through data indexing and encryption strategies that are not a focus of this paper. The participant was advised that the content would not be shared with anyone beyond the research teams.

The framework goals guiding this initial design work on an MHMR platform are illustrated in Figure 1. Patient Curation of Private Data (created as short duration first-person videos, which can contain private opinion and reflections-qualitative data, as well as measured vital statistics-quantitative data—that are owned and curated by the patient) leads to Process/Activities: Private Data Driven Option Identification/Evaluation (Patient generated data used by patient with possible input from trusted counsellors including Health Care Providers (HCPs) as evidence to develop knowledge, attitudes, and beliefs concerning care and accommodations options accessible through the patient's circle of care). These Input and Process/Activities may ultimately lead to Output: Acceptable Collaborative Care (supporting bilateral communication and collaboration between patient, HCPs, and circle of care in making decisions around care use options). Our model attempts to illustrate how Inputs from individual experiences can affect Processes and lead to positive and negative Outputs. The dotted line in Figure 1 represents the focus of this paper.

## Methodology

This study was approved by the Toronto Metropolitan University's (formerly, Ryerson University) human research ethics board (Protocol # REB 2016-150). The study followed all necessary procedures to ensure the confidentiality of a participant. Prior to the study, the participant co-designer completed and signed a written informed consent document. Personal information was then gathered.

The methodology employed in this research is a combination of an exploratory case study method (26) and the participatory design method (27, 28). The first stage of the system under investigation is the creation and storage of short-duration videos created by an individual with later stages being to support the communication and knowledge transfer between HCP, circle of care and the individual enabled by the MHMR application. The exploratory component arises as no specific outcome is expected. Rather, we want to explore how the individual will use the system, what topics are addressed and the content of the videos.

It is expected that the trajectory of results will identify meaningful and measurable outputs over time and can be instrumental in generating outcomes as the study proceeds. The research is carried out in natural environments where the participant passed time at home, school (workplace) and social settings. All recordings made by PX are converted into MP3 files and distributed among the research team for coding into themes and major domains.

Participant Co-Designer and System Use (identified as PX in this document with no relationship to the individual's actual initials or gender identity). As a first step in the system design, we recruited a co-designer participant to assist in developing a usable platform. PX played a pivotal role in shaping the MHMR design through active participation in the development process. This encompassed contributions to brainstorming, ongoing feedback, validation of design choices, user-interface problem-solving, and direct involvement in usability testing, all of which enriched the project.

In 2015, PX experienced the sudden onset of an arthritis-like inflammatory condition with mysterious origins. This resulted in multiple motoric disabilities, including limited use of legs and hands. After a 3-month hospitalisation that was unsuccessful in diagnosing a cause and providing a treatment strategy, PX was discharged home to other formal caregivers (physiologists, rheumatologists, etc.) and, with the support of PX's family, expected to find ways to cope with this new situation. PX's family was known by one of the researchers and was contacted to invite PX to join the project as a participant co-designer. PX agreed to use the MHMR application on a tablet version over a three-month period to help us understand the usability of our initial implementation of the MHMR platform and the extent to which interacting with it was experienced as meaningful and useful. PX began using the system during a 3-week business/pleasure excursion soon after leaving the hospital. Once returned, PX used the system at school for 11 weeks.

In total, PX recorded 65 videos with an average length of 36 s (s) (ranging from 12 to 148 s). Apart from being informed that the ultimate purpose of the technology was to allow people living with chronic conditions to document, reflect and comment upon their condition, PX was not given any direction on topics or times for creating his videos. Nevertheless, she focused on physical pain, difficulties or successes in daily activities, overall frustrations or other events that required attention. During the three-month study, PX provided continuous feedback and ideation to the team via email or in person with unstructured comments of usability issues and on how the system might be improved. During this time, we also noticed that PX stayed engaged in the effort, preventing the project from being abandoned.

## Qualitative analysis

The Consensual Qualitative Research (CQR) methodology was selected to analyze the qualitative data as it has been shown to be effective to better understand complex data and reducing bias that can result with a single researcher (29). The CQR updated method proposed by Hill, Thompson and William (29) is an inductive, open-ended research method for investigating unobservable internal experiences carried out through consensus between two invested researchers. It allows researchers to remain open to the data's revelations while focusing on and discussing participant narratives and descriptions together. This method allows for in-depth investigation of occasional events and emergent outcomes, making it useful when knowledge in a specific area is limited. It promotes diverse perspectives in order to have a thorough picture of the phenomenon (29). Additionally, team members were able to discuss disagreements respectfully while agreeing on themes that best captured the meaning of the data. The videos generated by PX were open-coded for relevant and recurring themes (see Table 1).

**Table 1:** Mapping of domains, themes, and definitions that evolved from PX's video content.

Domain, themes	Definition
Person facing: (outputs)	Physical and mental experiences related to the individual.
Pain, exhaustion, stress, Irritated, frustrated	The individual is in a state of physical and mental pain, distress, and suffering
Joy	Delight or happiness that the individual feels about an experience
Therapy/physio	Training or therapy to help rehabilitate and allow the individual to cope
Accessibility experiences: (processes)	Positive or negative comments and emotions, actions taken by others, or systems related to accessibility.
Environmental	Tangible or concrete situations or factors that affect the individual
Recommendation	The individual expresses their thought process and observations or comments about the incidents
Technical	Comments about technology and the MHMR application
System facing: (structure)	Descriptions/comments about physical, communication and attitudinal experiences and barriers experienced in various settings.
Culture	Ideas, customs, and social behaviour of a society different from that of the individuals
Communication	Professional interactions with people

The first step in the analysis was to formulate domains and themes. Two independent reviewers reviewed the videos without communication with the other team members. These reviewers coded blocks of video data (phrases, sentences) into themes, and labelled and defined them. Upon individual completion, all team members rejoined and discussed the themes as peer reviewers. Through discussion and consensual agreement, three domains and eight themes were created to capture all data. Tables 1, 2 provide the domain and theme labels, their definitions, and examples from the data of each theme. Furthermore, Table 1 shows how each domain fits within the SPO framework.

**Table 2:** Themes and quotations.

Themes	Quotation
Pain, exhaustion, Stress, irritated, frustrated	A “Today was the first time (Physiotherapy) had a 9 am session….worst decision ever, my body was so stiff and I got drained out, it was too difficult for me" B “along with school another thing is very stressful is a recruitment process as it’s my final year so it's so much pressure and I have to apply everywhere and have to get the job offer as its a last kick of the can.”
Joy	A “visit the conference… Great sets of lectures especially D… it was so amazing, so inspiring, motivational, charismatic and great to listen to him whereas others were fine” B This morning I went swimming………, and it's pretty cool because it was hot water, the hot tub was very relaxing, especially for muscles, …”
Therapy/physio	A “I went to physio yesterday and my physiotherapist was quite impressed by my progress, ……….., slow and steady,…"
Environmental	A “The opening ceremony was great stuff and the people were very accommodating…the whole day I was accommodated which was very fun…. I got around pretty nicely……” B “The weather in Denver is horrible in the sense that its the same as Toronto, it snowed a lot, and no one cleaned it up. Snow on the sidewalk the road everywhere so hard to walk I was slipping and off balance, I didn't expect that,.”
Recommendation	A “I wanted to talk about my evening class where we are talking about the wheelchair manufacturer industry and my professor kept on putting me on the spot, which was not nice, …….he doesn't have to put me on the spot like what does PX feel like…I didn't get a chance to speak to him but for sure going to follow up to him on email for this.”
Technical	A “…This is the third time I am recording this, hopefully, the camera doesn't shut off and this gets posted.”
Culture	A “Today is Thursday the learning journey was pretty cool and went to a local school in Hanoi, Vietnam and donated about … bucks, ………, students didn't see outsider (often), all the students were handpicked across Hanoi public school and it was pretty cool,..” B “So Sydney has been treating me great, love it, very accessible, very accommodating, but too many hills and it's tough getting around ….. ………”
Communication	A “So it was a pretty interesting event in yesterday class, so my professor was talking,, explaining and this was in the beginning of class so I respectfully had my hand up for a while then he's like you have your hand up for a while and there's nothing to be discussed, ……and I m just like yeah…’ can you just speak louder’ and, he said ‘I can’t that is the loudest I can speak and I said ‘ I cannot hear you’, so he said ‘come to the front’ pretty much, …I was shocked….., and I said ‘but I can't’ and he said ‘well I guess we’re stuck we can't do much about it’ “ B “So I got a job offer with (company) and they have acceptance party, and the best part about this is that they called me up and asked me if I'm comfortable with the venue, and if not they will change the entire venue…”

## Validity and reliability of analysis

As recommended (30, 31), several quality criteria were used to ensure the validity of the results and their interpretation: two independent coders with different backgrounds coded 20% of the data-set to ensure the reliability of the themes and definitions A Cohen's kappa coefficient was calculated. If kappa was below 0.6, an iterative process was then performed to understand and mitigate the differences. All of the themes and definitions contained in Table 1 had Cohen's kappa greater than 0.88. The remaining data were then coded by a single rater.

## Results and discussion

In general, PX was satisfied with the technology even though there were some technical difficulties associated with the tablet-based implementation. He was able to record short-duration, first-person videos, in several locations, that captured her ideas and topics. In addition, although she recorded videos about numerous topics, these tended to be centred around her pain experiences and related disabilities. There were no videos related to other topics such as family, friends, restaurant reviews, etc., that were independent of his pain or related disabilities despite explicit instruction to use the technology for whatever purposes seemed appropriate. This reflected perhaps his understanding that the ultimate goal of this research was to create a medium for sharing personal experiences related to the disability between interactions with members of the user's circle of care. In addition, the participant was enthusiastic about the research goal of finding ways of using this evidence to improve the experience of others facing disabilities due to a chronic condition of sudden onset. The videos are unlike the types of public contributions typically seen on social media and were more private and reflective in nature. There were no indications that PX found the process onerous or boring, and use actually began while PX was travelling on an extended multi-week trip. The total number of videos per domain were: Person Facing (43), Accessibility Experience (29) and System Facing (26) for a total number of videos equalling 65. The number of statements and videos containing specific themes were illustrated in Table 3 (some videos contained more than one theme).

**Table 3:** Frequency of statements and appearance in number of videos for each theme.

Domain	Themes	Number of statements (% of comments for domain)	Number of videos containing comments from theme (% of total # of videos)
Person facing (82 total comments)	Pain, exhaustion, stress, irritated, frustrated	57 (70)	49 (75)
	Joy	19 (23)	18 (28)
	Therapy/physio	6 (7)	6 (9)
Accessibility experiences (76 total comments)	Environmental	46 (61)	33 (51)
	Recommendation	28 (37)	28 (43)
	Technical	2 (3)	2 (3)
System facing (52 total comments)	Culture	14 (26)	9 (14)
	Communication	38 (63)	30 (46)

**Domain 1:** Person Facing:

(1) Pain, Exhaustion, Stress, Irritated, Frustrated: This theme had an overwhelming majority of comments and videos containing expressions from this theme among all of the themes in the person-facing domain. PX described or commented 57 times of the total 82 comments in the person-facing theme (70%) in 25 of 43 (58%) person-facing videos and 49 of the 65 total videos (75%). For example, PX describes the physical pain during a conference: “I just couldn’t take it going the whole day. It's very painful.” PX expressed and characterize her pain in several negative and positive emotional dimensions such as lack of sleep, anger, frustration, as well as hope or joy (see Theme 2). For example, “I’m not getting enough sleep for the past two-three days and, on top, have pain in the knees and ankles sore…”

This kind of feeling and emotion characterization is consistent with other studies (32). In addition, it is not surprising that the majority of video scraps are related to this theme. The idea that pain can lead to feelings of frustration, worry, anxiety, and depression seems obvious, particularly if it is of a chronic nature (32, 33). It should be noted that chronic pain can also lead to long-lasting emotional disturbances often referred to as a “secondary pain affect” (34). Negative emotional responses or low mood states, such as anger or depression, can intensify the pain experience (35–37).

In particular, PX showed that he often expressed: feelings of confusion and worry by the pain, and the social unpleasantness of living with pain. Corbett et al (38), also found that people living with pain often express despair related to the lack of empathy by “others.” Examples from PX include: “In the evening, I didn’t really do much, I was just bored in the room, my roommate went out.”

Although PX received no instruction or direction on topics for videos, ones related to pain were dominant. This may indicate that the documentation of pain experiences was a priority for PX or that she was comfortable sharing such videos with the design and development team. Identifying and tracking instances and frequency of pain, exhaustion, stress, irritation or frustration may allow users to reflect on when these emotions occur and examine trends of sustained negative emotional states. This may, in turn, lead to new ideas, solutions or coping strategies.

Keeping pain journals or using pain scales on a long-term basis is usually encouraged by HCPs to document and track pain experiences (39), but often, these are abandoned or seen as not useful to the individual or the HCPs (40). In addition, there can be considerable quantities of data generated, which must then be synthesized and organized into meaningful information. Having more creative and non-text outlets may improve people's ability and interest in long-term documentation and tracking.

(2) Joy: Not all expressions related to pain experiences were negative particularly when PX was hopeful of change although there were considerably fewer expressions of joy than pain. Of the 19 expressions of joy (23% of the total person-facing comments), 9, 7, 3 occurred when he was involved in personal activity, conference and classroom respectively. Prior research on positive expressions of happiness, inspiration and emotional well-being is limited as most of the documentation processes employed in pain have been associated with measuring or document pain exclusively (41) rather than the wide range of emotions that are seen in PX's data. Positive affect, according to numerous theorists, facilitates approach behaviour or continued action (42). From this perspective, experiences of positive affect could prompt individuals to engage more or earlier with their environments and partake in activities.

For example, PX shared his activities,

“This morning I went swimming with my brother, there was no else in the swimming pool and it’s pretty cool because it was hot water, the hot tub was very relaxing, especially for muscles, … I had the first time at the rehab, so this was a good start and relaxing after the whole week of hectic travelling”.

“First day back to school was amazing because the professor teaching us the audit was just amazing, he simplified everything to the take made it like, he called it the grandma language if you can explain it to your grandma then it makes sense otherwise it’s not. So it was so fun, (able to engage) it was nice, mind-opening and mind-blowing.”

He was able to record that his ability to participate in pool activities, the classroom and the conference demonstrated that an individual's own mental state such as joy, relaxing and positive anticipation could be a factor in the experience of pain. A next step would be to link factors such as the frequency and intensity of negative and positive emotions with pain or related experiences as well as their proximity to each other. For example, how many positive experiences are required to offset the negative emotions elicited from pain, and how does the time interval between positive and negative emotions mitigate the experience of pain and/or associated issues such as long-lasting emotional disturbances?

(3) Therapy /Physio: There were only 6 comments of the 82 person-facing comments (7%) with 9% (6 of 65) of the total number of videos containing comments from this theme. For successful therapeutic encounters, PX described feeling a sense of security and belonging, or expressed a sense of empathy and engagement. For example, “I went to physio yesterday and my physiotherapist was quite impressed by my progress…and that's why he still kept me on his caseload.”

PX gave positive accounts of his experiences as a patient, which often contextualized the service as an interpersonal relationship in primary or tertiary services. Blockley et al. (43) found that supportive interpersonal relationships reduce patient vulnerability and that nurses play a key role in the development and maintenance of these relationships. Research conducted on the experience of physiotherapy in rehabilitation services reported finding one main theme of personal interactions, and five sub-themes (empathetic and caring physiotherapist, socialisation with other patients, alleviated boredom, changed perceptions of the weekend, and contentment with the amount of therapy) emerging from their data. Patients valued interacting with physiotherapists and other patients (44).

For example,

“Today I have done too much physio lately [with the assistance of the staff] and my knees hurt it was a different pain now, they seem weak but yeah it hurts same old …..I have to figure it out, pretty much.”

In addition, having a feeling of mutual understanding and recognition by staff enabled PX to move toward acceptance of his pain as something that was part of his daily living. These factors (personal relationship and understanding) appear to be more important to an individual with pain than the amount of therapy received (44).

**Domain 2:** Accessibility Experiences:

(4) Environmental: The environmental theme in the accessibility domain constituted the next most discussed theme after pain in the entire video set. Fifty-one percent of all videos (33 of 65 total videos) contained comments related to the environmental theme.

In the environmental theme, comments were related to physical infrastructure, weather, the physical location of people around her, and the accessibility services. Positive comments included enjoying his time at an event, feeling accommodated, being independent and respected and enjoying the company of others.

For example, during a conference PX recorded a positive video about the physical accessibility offered by the hotel “after registration we got the room keys and I got the accessible room, which is pretty big and spacious, with a double bed and shower chair. Very nice, very accessible.” A second example concerned a positive experience with the availability of accessible airport services: Denver airport was pretty good we landed late night at 12 am something and it was a long walk, so they offered the golf car and a wheelchair was also available, there were no long lines (for me, for immigration) …not to wait so it was great.”

Poor accessibility for PX in the environment also occurred and resulted in negative expressions including feeling out of control, stressful, angry, alone, dependent, or disorganized. In particular, PX expressed frustrations with inaccessible physical environments or lack of accessible resources especially those that made her reliant on others for assistance. E.g.,: “Oh my gosh! Z (institution) needs to fix all the doors, those automatic doors are not working like you know first of all they’re so heavy so I cannot push them and the people are so inconsiderate they see me struggling and I am kind of pushing it, but they still don’t come by to help me, I don’t know which world they’re living in, but like that's pretty rude I would say but I guess they just don’t care, so I got to deal with it. It sucks when it's nighttime after my 10 pm class there is like no one, the whole campus is dead so there is no one to help me.”

In the social model of disability, the three main barriers experienced by people with disabilities (PWD) are environment, attitudinal and systemic in their environment (45). Physical barriers refer to obstacles in the built environment (e.g., architectural, transportation, communication, services, and physical infrastructure) (46), attitudinal to attitudes towards PWD by others (47), and systemic barriers introduced by a system that would include barriers from policies, procedures, legislation, cultural values (e.g., by government, and organizations (48). PX identified and experienced all of these barriers and, similar to comments in the pain and therapy themes, realized that he was facing a need to “deal with it on my own.” As a result of PX's sudden onset of a chronic condition, her experience with barriers was also sudden and novel. Neither he nor his parents had any experience with the disability community, self-advocating, or available resources. Feelings of being alone and needing to address barriers independently are common occurrences among PWD (49). The process of learning to self-advocate and find resources can be challenging and is often frustrating, particularly for people with newly acquired disability (50, 51). Being able to record and reflect on experiences of barriers and challenges may expedite this learning process because evidence can be collected, reviewed and presented to other parties as support for requests, complaints, recommendations, or accommodations.

(5) Recommendation: Twenty-eight of 76 comments (37%) in the Accessibility domain were related to the Recommendation theme. They consisted of opinions, positive feedback, and offering suggestions about coping or accommodation strategies for institutions, individuals and events. These recommendations were particularly evident in comments about PX's educational institution. An example of a recommendation for an individual was:
  “ In my evening class where we were talking about the wheelchair manufacturing industry and my professor put me on the spot, which was not nice……he can generalize and make statements, he doesn’t have to put me on the spot … I have tried to speak to him but didn't get the chance I will follow up on the email.”

An example of a recommendation for an event was: “I have a presentation in a week and I am just thinking about how it will go because there are stairs to get to the front, so even though someone clicks the slides would it still be the same thing if I speak from the back of the class, would that be the same as presenting from the front and how would that affect my presentation, and my marks”.

Finally, an example of a suggestion for an institution was: “So I am actually debating, arguing with another professor too about another mark, participation mark. The way he marked, ‘participation mark’ is so weird that I am just like this doesn’t make sense, so we’re going back and forth, …………there is no point in arguing with him. So I am just waiting to get my final participation marks and take it to the director of the school or the UPD and discuss it with him.”

It is well known that with some exceptions (e.g., McCloy and DeClou 2013) (52), few recent publications consider educational accessibility for a diverse spectrum of students who are identified as disabled in Canadian institutions (53). In addition, Rosenzweig (54), suggests that special training in which faculty, general, and accessibility staff must be educated about behaviours and disabilities strategies that could be used to support more inclusive classrooms and education. However, as discovered by PX, few instructors seem to have knowledge of these strategies and how to apply them in the classroom. Being able to record a situation and discuss the resulting impact on the individual may assist individuals in presenting evidence to concerned parties. It may also allow an individual to reflect on their reaction to a situation and consider appropriate actions at a later date or based on their own suggestions.

(6) Technical: There were only two comments (3%) in the Accessibility domain about the recording system and technology. Both comments related to the recording system failing to record or stop, and having to re-record the session. In this case study, we focused mainly on the first stage of the journey (building confidence in the documentation) while being mindful of the full journey. Consequently, the system used by PX was an early prototype where we wanted to first understand the process of and ability to record short videos. We also wanted to understand the types of topics and commentary that were generated. Despite experiencing some technical difficulties, PX was keen to document. For example: “Oh my gosh! okay this is the third time I am recording this and I want to talk about my evening class and its incident.”

The technical difficulties experienced by PX assisted the research team in correcting errors and improving the system.

**Domain 3:** System Facing:

(7) Culture: This theme represents comments about customs and traditions in other countries related to access; these include comments on transportation, scheduling, out-of-pocket costs, and resources. In the system-facing theme, there are 14 of 52 (23%) comments). Attitudes towards disability are not always uniform within a region or even within a country. Different groups or individuals may have beliefs about disability that vary from those held by the wider society. Beliefs may also vary even within small communities and even within families.

PX experienced support (or lack of) from the same or different systems, often expressing positive and negative emotions associated with those experiences. For example, in Japan,

“Japan was just amazing we got escorted out the doors,.…..The washrooms were very accessible, has a bed there as well… when we took the train, the train staff would escort me to the platform and took out a ramp and got me in.. And the best part……they already sent a message and the person was standing there, just waiting, it was mind-blowing and so accommodating….. streets were amazing flat straight, we went out into the city, it was very nice, loved it,”. “ I’m trying to board for Hanoi from Japan, which was pretty frustrating because they didn’t allow to me to take the wheelchair to the gate, there like I am going to get my wheelchair with the luggage pickup, that was pretty annoying.”

Since the United Nations adopted the Convention on the Rights of Persons with Disabilities, ratified by 168 countries, there has been progress as well as stubborn obstacles (55). PX encountered the results of efforts to improve accessibility as well as the obstacles that have yet to be rectified. Having a way of capturing the instances of those barriers may offer individuals like PX with support in advocating for improvements on a small scale. Collectively, if many narratives can be gathered together by more than one person, PWD or their associates can provide evidence to justify demands to communities, governments and/or organisations for improvements and resolutions for change.

## Communication

The final theme captures PX's desire to express his personal stories of the interaction in hope of it positively influencing others. PX articulated the need for ethical and inclusive environments at the institution. However, most of the 38 comments (14) in this domain were negative where PX expressed disappointment, frustration, struggle and a desire to be treated fairly and with dignity.

e.g., “I got an assignment back and got a 70% which I think I had done better or would’ve done much better but I wasn’t able to attend one of the classes due to an [medical] appointment. I told the professor and he didn’t have any slides for that class. He tried to gather some notes and by the time he gave me the notes it was the day when the assignment was due and I submitted and then I saw the notes so it was too late to incorporate them…so I emailed the prof and let's see if he gets back to me. Technically speaking I should’ve been accommodated if someone has an appointment and does not have the slides just that day… no one takes notes in that class.

For e.g., “Oh my god! So it's more than a week and since I have reported to maintenance for the doors (to repair) and still haven’t fixed it”.

While these examples and most of PX's comments in this study are common and congruent with others (e.g., 45) they were new for PX. Being able to document these experiences may offer personal benefits such as having a mechanism for “venting”, reflection and, perhaps, mediation. In addition, finding ways to automatically analyze the video material may offer opportunities for discovering patterns, topic threads or specific issues. Finally, if these videos can be collected together with others, they may be used to affect systemic change by providing evidence of patterns and ongoing, common issues.

## Summary discussion

PX was able to successfully make recordings and capture her successes, failures, issues and concerns on a number of different but related topics using the MHMR technology. The MHMR interface appeared to have helped PX's need and want for chronicling an assortment of topics including ongoing barriers, hindrances experienced, dissatisfaction, pain, and excitement. Despite no enforced video length cut-off, brief recordings were recommended and seemed to be preferred by PX. Constraining recordings to as short as one minute would be a possible interface for the MHMR system and may simplify any large-scale application of the approach because it is easier to store and manage short videos compared with larger ones.

Another interface issue was allowing the deletion and re-recording of a video related to a specific domain. PX suggested that a video generated in a single take was more genuine. It is possible that others may find it difficult to make a point in a single take, particularly for novice users who must learn how to use the medium. However, it would be up to the user to decide what is shared regardless. It is unclear whether PX was suggesting that his recordings became inauthentic as a result of multiple takes or whether others may judge her recordings as inauthentic from a more polished appearance and flow as a result of the practice offered by multiple recordings. Future research will examine the effect of multiple recordings on the assessment of trustworthiness, believability, and authenticity by the individual as well as those with whom the recordings would be shared. There is likely to be a trade-off between the view of authenticity, genuineness and the clarity and efficiency that comes with practice. Further research about restricting or reducing the number of takes and the effect on authenticity and exertion is required.

The second research question addressed the possible topics that were recorded by PX and whether those reflected his current life situation. No instructions or direction was provided to PX about which topics to use in order to give him control and flexibility over them. However, PX was recruited by the research team early on so he was aware of the purpose of the project which may have indirectly influenced topic selection. While there was a wide variety of topics that were broached by PX, the majority of them were related to her pain and experiences as a person with a disability. In addition, these topics could be categorized into domains related to the self or inward-looking, those related to a system (e.g., education, transportation, and government) or outward-looking, and to accessibility or barriers experienced using a standard qualitative evaluation method. Technical aspects of the MHMR system was the only topic not related to pain or disability.

PX had newly acquired the status of a person living with a major chronic disability. That may have influenced his perspective and experiences as they were all new to her. Involving people with either congenital disabilities or who have been living with a disability for a long time may show different results regarding the level or number of comments related to accessibility or barriers. However, people can experience disabling conditions suddenly at any point in their lives. MHMR can allow people to document those experiences for reflection or to serve as a simple method of collecting evidence for future treatment, advocacy or service acquisition.

### Limitations

There are a number of limitations of this study; first and foremost is that it presents results from a single case study involving a self-selected, educated individual with newly acquired chronic pain and associated disabilities. A larger sample with a variety of people with chronic illness and pain will need to be recruited in order to make generalisations about the use and usefulness of MHMR. However, it was important to determine whether it would even be feasible to launch, support and use MHMR in the “wild” in a longitudinal context as part of the formative work that is necessary to justify future work.

A second limitation was that all files were encrypted once PX clicked the save button, for security purposes, and all date-related information was lost. This reflected using open encryption platforms and their default privacy configurations compatible with the Android devices used for this simple usability phase of the design process, rather than the comprehensive data registration service developed by gDial Inc. that is now being adapted to operate on Android systems. As described by Pennefather et al (8), there is a need to save as much contextualization information concerning the videos as possible to increase their value. This will require the creation of personally controlled data accounts to serve as private data repositories whose entries can be made selectively public for specified limited purposes (8). As such, we could not determine when specific recordings were made to examine any novelty effect of the MHMR system use. However, the frequency of MHMR recordings may not be related to novelty but instead to the salience of specific situations. The current iteration of MHMR has resolved this issue.

Another limitation was that we only examined the input stage of the MHMR system. We did not examine the curatorial process or output/sharing components of MHMR. It was important to determine whether the data-collecting task was feasible over the long term before efforts were made to complete the system. Written journals and diaries are common methods for collecting a patient's thoughts and feelings about their health situations (56). However, the abandonment rate is high (40) and many studies do not carry out longitudinal studies. We were interested in determining the feasibility of using the MHMR system to capture a patient's thoughts and feelings over a longitudinal time-frame as a first step in designing and developing the entire system described in Figure 1. Furthermore, the study does not explore topics such as digital literacy, inclusivity, and socioeconomic difficulties in the setting of chronic disease as potential influences on the outcome, however, these topics will be a focus of future research with additional participants.

Although there are limitations in this formative research, we believe that this study adds to the growing body of knowledge on digital and visual reporting and how it may play a role in addressing the needs of individuals living with chronic pain or illness. The project hypothesis, that communicating the experience of illness through recording and registering video commentary in a video scrapbook format may provide positive psycho-social benefits to some patients with chronic pain or illness, appears to be supported.

## Conclusion

The paper presented two related notions: (1) to describe the experiences and insights of an individual with chronic illness in their day-to-day life, and (2) how technology could assist in addressing or overcoming the disability associated with that experience. A single perspective was explored: a person with a new but chronic pain condition who was learning to self-manage his condition and document that process with the technology provided. Using MHMR as a personal self-reporting diary/repository over a three-month period showed that it was feasible to generate ongoing and valuable insights over the longer term, as evidenced in the pilot case study. Moreover, themes could be identified that usefully could be distinguished in terms of patient facing (perceived consequences/outputs).

The next steps are to build a seamless and efficient communication channel that allows individuals with chronic illness, healthcare professionals, and other stakeholders to access, evaluate, and act on acquired data, fostering a collaborative approach to care. We aim to employ artificial intelligence and data visualization techniques to analyse, summarize and illustrate the collections of the recorded qualitative data. This will involve developing interactive visual representations that summarize the data into meaningful collections and interpretations of the subjective/qualitative information, and then provide means to share those summaries with an individual's circle of care. It is also important to understand and evaluate how communication between a patient and their circle of care can be facilitated and whether that communication facilitates improved health and well-being outcomes.

To enable the appropriate use of such data in healthcare, we envision a system in which the collected information is securely and easily accessible to care practitioners and other stakeholders. Implementing a safe data-sharing method that conforms with all relevant data privacy standards will be a part of our recommended solution. Future studies will require testing the application with a larger group and integration of qualitative data with quantitative data available from other sources like wearable devices or patient portals.

## Data Availability

The datasets presented in this article are not readily available because the data will not be available in a public repository due to the participant's privacy and confidentiality issues. Requests to access the datasets should be directed to SFL.
